# A Case of Heterotopic Pancreatic Tissue Discovered in the Distal Esophagus

**DOI:** 10.1155/2020/4695184

**Published:** 2020-04-09

**Authors:** Dema Shamoon, Vanessa Sostre, Varun Patel, Ariy Volfson

**Affiliations:** ^1^Department of Medicine, St. Joseph's University Medical Center, Paterson, NJ, USA; ^2^Division of Gastroenterology and Hepatology, St. Joseph's University Medical Center, Paterson, NJ, USA

## Abstract

Heterotopic pancreas (HP) is a congenital abnormality that represents ectopic pancreatic tissue that does not have anatomic, vascular, or ductal continuity. The prevalence of HP is 0.55% to 13.7% on autopsy, 0.2% to 0.5% of abdominal operations, and 0.9% of gastrectomies. It is commonly found in the stomach, duodenum, and proximal jejunum. Only 15 cases have been reported in the medical literature regarding involvement of the esophagus. Treatment depends on symptoms and location. In asymptomatic patients, simple observation may be sufficient; however, in those who are symptomatic, surgery may be warranted. We present a case of a 70-year-old male with heartburn, nausea, and abdominal bloating who underwent a diagnostic esophagogastroduodenoscopy (EGD) and was found to have HP on histology in the distal esophagus. In our case, symptoms were treated conservatively and successfully with a proton pump inhibitor (PPI).

## 1. Introduction

A heterotopic pancreas (HP) is a congenital anomaly that is anatomically separate yet histologically the same as the pancreas [1, 2]. This anomaly is also known as ectopic, aberrant, or as an accessory pancreas as it does not have anatomic, vascular, or ductal continuity [1, 2]. Cases are most commonly seen in the upper gastrointestinal tract (GIT) such as the stomach, duodenum, and proximal jejunum [2–4]. Although less common, HP may also be seen in the esophagus, ileum, Meckel diverticulum, and biliary tree [2]. HP affects males two times more than females and typically is not discovered until the fifth to sixth decade of life despite a few cases in children and young adults [5]. HP presenting in the esophagus is uncommon with approximately fifteen adult cases reported in the medical literature [1, 3, 5–19]. We present a case of a 70-year-old male who was incidentally found to have HP located in the distal esophagus on EGD after reported complaints of heartburn, nausea, and abdominal bloating.

## 2. Presentation

**Figure 1:**
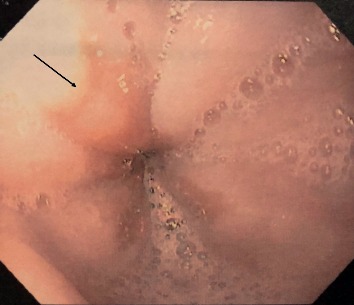
Endoscopic image of a polypoid lesion in the distal esophagus above the gastroesophageal junction.

**Figure 2:**
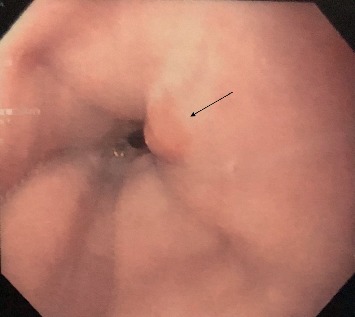
Another endoscopic image of a polypoid lesion in the distal esophagus above the gastroesophageal junction.

**Figure 3:**
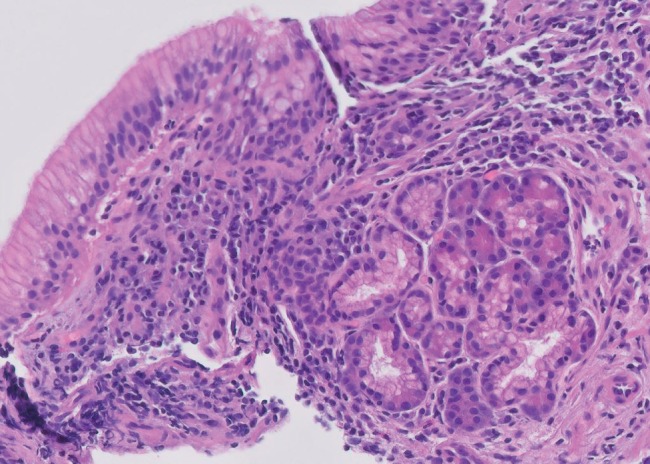
Histopathology of the distal esophageal submucosal lesion showing a well-circumscribed small nodule of pancreatic acinar glands.

**Figure 4:**
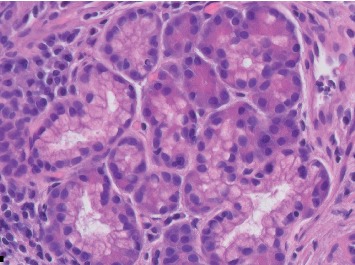
Histopathology of the distal esophageal submucosal lesion with higher magnification showing a well-circumscribed small nodule of pancreatic acinar glands.

**Figure 5:**
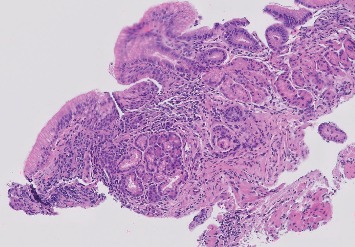
Histopathology of the distal esophageal submucosal lesion at low magnification showing a well-circumscribed small nodule of pancreatic acinar glands.

This is a case of a 70-year-old male with a medical history significant for hypertension, hypercholesterolemia, melanoma, and atrial fibrillation who presented with complaints of heartburn, nausea, and abdominal bloating. Patient reported to also have hiccups along with nausea but no episodes of vomiting. Patient endorsed mild abdominal distention. He denied abdominal pain, hematochezia, melena, hematemesis, change in bowel movements, or weight loss. Physical exam did not reveal any abnormalities. Routine blood work was unremarkable. Initially, our patient was treated for dyspepsia with a trial of PPI. He was scheduled for an EGD, which revealed an incidental 5  mm polypoid appearing lesion in the distal esophagus above the gastroesophageal junction (Figures 1 and 2). Biopsy results revealed squamous mucosa with active esophagitis and associated cardiac-type mucosa with focal pancreatic heterotopia and chronic inflammation in the distal esophagus (Figures 3–5). Given the relatively small size of the lesion, it was resected endoscopically. The patient was treated conservatively with supportive with PPI for his gastric inflammation and symptoms. Patient is to be followed in one year for surveillance of the lesion with biopsy. Patient may need an endoscopic ultrasound (EUS) depending on his surveillance EGD results.

## 3. Discussion

Heterotopic pancreatic tissue is an uncommon congenital malformation that typically is found incidentally due to its nonspecific nature [4]. The incidence is hard to determine; however, the prevalence of HP is 0.55% to 13.7% on autopsy, 0.2% to 0.5% of abdominal operations, and 0.9% of gastrectomies [2–4].

The pathogenesis of the pancreas involves the fusion of the ventral and dorsal outpouchings of the foregut with detachment of branching pancreatic buds [3]. The embryologic basis of HP is unknown, but the theory of misplacement describes how pancreatic tissue is deposited in developing areas of the GIT [2]. Two additional theories are described in the literature [2]. The metaplasia theory depicts the migration of endodermal cells to the mucosa and subsequent change to pancreatic tissue during the time of embryogenesis [2]. The totipotent cell theory involves differentiation of cells that line the gut [2].

While HP does not have anatomic, vascular, and ductal continuity to the pancreas, it may still function similarly if it consists of acini, ducts, and islets of Langerhans [3, 15]. Acini, which are responsible for the exocrine function of the pancreas, permit the secretion of enzyme-rich serous fluid into the duct lumen due to its membrane-bound zymogen granules [3]. Leakage of proteolytic enzymes may lead to tissue breakdown and activation of the coagulation cascade [5]. Consequently, this event could lead to formation of small fibrin thrombi and secondary ischemic damage [5]. A secondary inflammatory response occurs from the release of lipase and phospholipase causing fat necrosis [5]. This is compounded by the release of proteases that digest protein substrates and further worsen the inflammatory response [5]. As seen in our case's histopathology, the chronic inflammation of the distal esophagus places him at higher risk for development of an inflammatory mass [5]. With the persistent inflammation and release of enzymes from cell breakdown, it may similarly present as chronic pancreatitis. Possible complications of ectopic tissue include benign or malignant transformation of tissue, pseudocyst or cyst formation, pancreatitis, gastrointestinal bleeding, ulceration, gastric outlet obstruction, or intussusception [2, 3, 15].  Table 1 exhibits the cases of HP found in literature with specific characteristics such as age, sex, location, presentation, and complications as well as treatment and follow-up.

Differentiating malignancy and HP can prevent unnecessary surgical intervention [2]. Most of the information available is related to HP in the stomach. Three criteria were reported by Guillou et al. that explain how malignancy may result from HP [8, 19, 20]. The first criteria states that the tumor must be found within or close in proximity to the lesion [8, 19, 20]. The second is when a clear evolution is seen between the carcinoma and pancreatic structures such as ductal cell dysplasia or carcinoma in situ [8, 19, 20]. The last criteria states that the nonneoplastic HP includes fully developed acini and ductal structures [8, 19, 20]. Our histology did not show malignant morphology and mainly pancreatic acini. The risk of pursuing surgical intervention immediately outweighs the risk of observation in this patient. The risk of malignancy from HP is rare and rather than subject an elderly patient with comorbidities to surgery, yearly observation seems appropriate [8]. Our patient presented with nausea and hiccups, but no other symptoms alarming for malignancy. If our patient develops symptoms of dysphagia, obstruction, upper GI bleed, pain, weight loss, nausea, or vomiting, it is important at that time to reassess as it could represent alarm symptoms for malignancy [2, 3, 5]. A feasible option is to also utilize EUS and fine needle aspiration (FNA) to further assess the depth of the lesion in comparison to the layers of the wall upon next evaluation. Of the fifteen cases of HP, two were found to be malignant and those patients had symptoms of weight loss, dysphagia, and epigastric pain [8, 9].

On CT, HP may appear as a small oval intramural mass with microlobulated margins and an endoluminal growth pattern [2]. Contrast enhancement and homogeneity are features that may apparel with histologic composition [2]. On MRI, HP is isointense to the orthotopic pancreas with characteristic T1 hyperintensity and early avid enhancement after administration of intravenous gadolinium-based contrast material [2]. Diagnosis occurs usually by EGD, EUS, operation, or autopsy depending on the presentation of symptoms and location of the tissue [4]. When visualized on EGD, a pancreatic rest may appear to be a submucosal bulge covered with normal mucosa with or without a central umbilication [16]. EUS may exhibit an intermediate echogram between the echodense submucosa and hyperechoic muscularis propria (MP) layer; relatively, the lesion appears to be hypoechoic to the submucosa and isoechoic to the MP [2, 16]. Others have suggested, such as Minamoto, that tubular or circular echoless structures may represent pancreatic rest [16, 21]. EUS also allows for sampling via FNA; however, it is not always specific to diagnose HP [2, 16]. Biopsy results are typically nondiagnostic due to the submucosal location of the lesion [10]. Histological evidence is usually achieved after submucosal resection and dissection of the area [10, 22].

Treatment of the condition varies based on symptoms and location. Options include observation, Ivor-Lewis esophagectomy (ILE), and surgical or laparoscopic resection [1]. Although many cases have shown that surgical resection is the primary treatment, conservative treatment when identified by biopsy may be possible as only few cases of malignant transformation have been reported [13]. In our case, this was an incidentally found lesion, and it was unclear whether it was the reason for our symptoms. Therefore, our patient was treated conservatively with one-year surveillance with EGD.

## 4. Conclusions

We present a 70-year-old male who was diagnosed by histopathology to have a heterotopic pancreas after complaints of heartburn, nausea, and abdominal bloating. This condition is rare with only about 15 adult cases reported. The lesion was resected endoscopically, and PPI was initiated for symptomatic relief as well as gastric inflammation. Patient will be followed with surveillance with biopsy with EUS pending biopsy results.

## Figures and Tables

**Table 1:** Reported cases of heterotopic pancreas in the esophagus among adults in the medical literature.

Case	Age	Sex	Location	Presentation	Treatment	Follow-up
Crighton and Botha [15]	58	F	GEJ	Progressive dysphagia due to IPMN	Laparoscopic/thorascopic ILE	Asymptomatic at 3 months
Temes et al. [10]	24	F	4.5 cm mass 3 cm proximal to the GEJ	5 days of nausea, vomiting, fever, chest pain, SOB due to esophageal rupture, and empyema	Esophageal enucleation, esophageal mucosa, and muscle closed	Asymptomatic at 1 year
Lowry et al. [1]	25	M	Mass located in submucosa of distal esophagus	RUQ and epigastric abdominal pain. EGD showed fistulous tracts 3 cm proximal to GEJ and stomach nodule	VATS resection	Asymptomatic at 2 months
Noffsinger et al. [5]	47	F	Distal esophagus	Epigastric abdominal pain, unable to tolerate solid foods, poor appetite, and weight loss for 2 weeks that was found to have 9 cm mass at the GEJ	ILE, pyloroplasty, Witzel jejunostomy	Infections and respiratory distress postoperative
Goto et al. [13]	63	M	∼2 cm in diameter submucosal tumor in the middle third of esophagus	Asymptomatic, incidental finding	Conservative management	Asymptomatic for 5 years
Ulrych et al. [17]	34	M	Tumor arising from the lower esophagus	Several years of dyspepsia with 3 months of progressive dysphagia, odynophagia, and regurgitation. Weight loss and weakness.	Left posterolateral thoracotomy, primary anastomosis, and partial fundoplication	Asymptomatic at 3 months
Gananadha and Hunt [12]	26	F	Mass located in the wall of the distal esophagus; caudal portion was involved with GEJ	Episodes of severe epigastric pain, occurring after food intake, and nausea	Diagnostic laparoscopy discovered mass in the wall of distal esophagus. Cephalad portion was cystic which was separated from esophageal mucosa. Caudal portion was excised using endo-GIA stapler. Partial Dor fundoplication performed afterwards.	Asymptomatic at 2 months
Roshe et al. [9]	45	M	Distal esophagus	Dysphagia for 6 weeks	Left thoracoabdominal esophagogastrectomy	Asymptomatic
Razi [7]	43	M	Distal esophagus	Massive upper GI bleeding	Thoracotomy for removal of the pleural over the esophagus; tumor was enucleated from the esophageal wall	Asymptomatic
Salo et al. [6]	25	M	Distal esophagus	Nonspecific upper abdominal discomfort, heartburn, and vomiting for 1 year preoperatively. 3 years postoperatively, EGD showed reflux esophagitis.	Intramural esophageal cyst was enucleated by right thoracotomy. Reflux was treated with metoclopramide and ranitidine.	N/A
Shalaby et al. [16]	52	M	Mass located at GEJ	Episodic dysphagia	Small food boluses	Asymptomatic
Guillou et al. [8]	60	M	Ulcerated mass located at GEJ	Epigastric pain, dysphagia, and weight loss.	Tumor resection by left thoracotomy with proximal stomach resection; esophagogastric anastomosis	Asymptomatic at first but then developed bronchopneumonia and died 3 months postoperatively
Rodriguez et al. [11]	41	F	Submucosal mass found at GEJ extending into lesser curvature of stomach	Dysphagia and epigastric pain	Total gastrectomy with Roux-en-Y esophagojejunostomy	N/A
Garn et al. [14]	38	F	Submucosal tumor of GEJ	GERD	Endoscopically assisted laparoscopic resection	Asymptomatic
Salim et al. [18]	29	M	Irregularity of Z-line in distal esophagus	Epigastric pain radiating to the chest, worsened by hunger. Dysphagia to solids.	N/A	N/A

GEJ: gastroesophageal junction; IPMN: intraductal papillary mucinous neoplasm; SOB: shortness of breath; RUQ: right upper quadrant; VATS: video-assisted thoracoscopic surgery; ILE: Ivor-Lewis esophagectomy; GI: gastrointestinal; GERD: gastroesophageal reflux disease.
